# Evaluation of an Outpatient Consultation Model for Cancer‐Related Fatigue by the Bavarian Cancer Society: Service Utilization and Patient Satisfaction

**DOI:** 10.1002/cnr2.70638

**Published:** 2026-08-23

**Authors:** K. Müller, I. Fischer, M. Besseler, M. E. Heim, J. U. Rüffer, J. Weis, T. Pukrop, M. Koller

**Affiliations:** ^1^ Center for Clinical Studies University Hospital Regensburg Regensburg Germany; ^2^ Institute for Tumor Fatigue Research Emskirchen Germany; ^3^ German Fatigue Society Köln Germany; ^4^ Working Group on Cancer‐Related Fatigue Within the Bavarian Cancer Society München Germany; ^5^ Bavarian Cancer Society München Germany; ^6^ Comprehensive Cancer Center University Hospital Freiburg Freiburg Germany; ^7^ Center for Translational Oncology University Hospital Regensburg Regensburg Germany; ^8^ Department of Internal Medicine III University Hospital Regensburg Regensburg Germany; ^9^ Bavarian Cancer Research Center Regensburg Germany

**Keywords:** cancer survivorship, cancer‐related fatigue, consultation, oncology care

## Abstract

**Background:**

Cancer‐related fatigue (CRF) is a common, debilitating consequence of cancer and its treatment, yet it remains frequently underrecognized and undertreated. As a result, many patients receive neither targeted assessment nor appropriate treatment recommendations. This study evaluates the utilization, intervention uptake, and patient satisfaction with a specialized CRF consultation service at 10 cancer counseling centers in Bavaria, Germany.

**Method:**

A prospective, observational, multicenter study was conducted between March 2022 and February 2024. Patients with a cancer diagnosis and clinically relevant fatigue were eligible for CRF consultation. Of the 557 patients who received a CRF consultation, 524 participated in the study, of whom 496 were clinically diagnosed with CRF; 447 completed the follow‐up.

**Results:**

Among patients with confirmed CRF (*n* = 496), a shared decision‐making process involving patient and clinician resulted in the selection of three evidence‐based interventions in most cases (80%), most commonly physical activity (63%), energy conservation and activity management (45%), and mind–body approaches (39%). At follow‐up (*n* = 447), 44% of the recommended interventions had been implemented either fully or to a large extent. Even for the most frequently selected interventions, implementation remained moderate (33% to 56%). Barriers to implementation—which were primarily related to health limitations, family and work commitments, and limited service availability—were reported by 87% of the patients. Despite these challenges, satisfaction with the consultation was high (mean value of 1.2 and 1.4, on a scale from 1 = very good to 6 = very poor), and patients expressed strong support for continuing the service. Suggestions for improvement included shorter waits, assistance in implementing interventions, and support with administrative procedures.

**Conclusion:**

The CRF consultation service of the Bavarian Cancer Society is a feasible, well‐accepted, and patient‐centered approach that addresses a major unmet need in cancer care. This model may serve as a best practice example for similar initiatives nationally and internationally. Long‐term implementation in the healthcare system requires structural integration, sustainable funding, and policy recognition.

**Trial Registration:** German Clinical Trials Register: DRKS00028264

## 1 Introduction

Cancer‐related fatigue (CRF), also known as tumor fatigue (TF), frequently occurs in patients with cancer as a consequence of or in relation to the disease itself or its treatment [1, 2, 3]. CRF is characterized by persistent exhaustion, weakness, and a lack of energy that considerably impairs daily functioning and quality of life [4]. Early diagnosis and treatment can substantially reduce the risk of chronicity [5].

The German Institute for Quality and Efficiency in Health Care (IQWIG) summarized national and international evidence‐based recommendations on CRF management, such as the identification of underlying treatable factors, provision of appropriate treatments and information material on CRF and its treatments, and referral to therapeutic facilities and support groups [6]. A gap exists between the availability of evidence‐based recommendations outlined in guidelines [1] and the actual practice of CRF management in current healthcare [7, 8]. Thus, CRF management is considered one of the major unmet needs in oncology care [9].

To address this unmet need, the Bavarian Cancer Society, a nonprofit organization operating cancer counseling centers across Bavaria, Germany, in cooperation with the Institute for Tumor Fatigue Research, has developed a specialized consultation service. This outpatient CRF consultation service was developed with reference to the Cancer Fatigue Clinic at the MD Anderson Cancer Centre [10, 11, 12]. The service was introduced in 2013 and gradually expanded to 10 regional counseling centers by 2021. The service follows an evidence‐based approach and is regularly updated to reflect current scientific guidelines [1, 11, 13, 14, 15]. The CRF consultation model comprises the following elements: (1) (differential) diagnostics, (2) identification of potential contributing factors, (3) individualized patient education about CRF, (4) collaborative selection of evidence‐based management strategies (interventions) within a shared decision‐making framework, and (5) provision of structured informational materials on recommended interventions. Depending on selected interventions and their availability, patients are either referred to services offered within the counseling service or directed to appropriate external providers (see File S1) [16, 17, 18].

The aim of this study was to systematically assess how patients utilized the CRF consultation service, the diagnostic procedures applied within the consultation, which interventions were selected through shared decision‐making, the extent to which these interventions were taken up by patients, and patient satisfaction with the overall process.

## 2 Method & Materials

### 2.1 Design

This prospective, observational, multicenter study involved two assessment points. Data were collected at the initial consultation (t1) and at a follow‐up 10 ± 2 weeks later (t2). Ethical approval from the University of Regensburg was obtained (No. 22‐2796‐101). The study protocol was published [19], and the study was registered with the German Clinical Trials Register (No. DRKS00028264). CRF consultation was provided according to the standard procedure at the Bavarian Cancer Society as described in File S1.

### 2.2 Recruitment

The CRF consultation was implemented in 10 cancer counseling centers of the Bavarian Cancer Society. All centers offering CRF consultation participated in the present study. Patients contacted their regional counseling center to schedule an appointment. Information about the availability of the CRF consultation was provided through the counseling centers, referring healthcare professionals, and publicly accessible information. All individuals who requested an appointment in the CRF consultation between March 2022 and February 2024 and met the inclusion criteria (fatigue/exhaustion, confirmed cancer diagnosis, residence in Bavaria) were eligible to participate in the study. Informed consent was obtained from the study participants. Patients who declined to provide consent were still offered access to the CRF consultation.

Based on the number of CRF consultations in previous years, we projected that 400 patients would attend the CRF consultation over a 2‐year period. We anticipated a follow‐up attrition rate of 10%–20%.

### 2.3 Data Sources

#### 2.3.1 Data Reported by Patients

Before the consultation, patients completed screening questionnaires to assess fatigue (Brief Fatigue Inventory [BFI] [20, 21]), depression (Patient Health Questionnaire‐9 [PHQ‐9] [22]), and anxiety (Generalized Anxiety Disorder Scale‐7 [GAD‐7] [23]). In addition, patients reported sociodemographic information (e.g., sex, age, highest level of education, employment status) and access pathway.

At the end of the consultation, patients completed a satisfaction questionnaire comprising an overall satisfaction rating (1 = very good, 6 = very poor), evaluations of 10 consultation‐specific satisfaction aspects (fully agree, mostly agree, rather disagree, and fully disagree), and a free text section for suggestions on future improvements [16].

At follow‐up, patients were contacted by counseling centers and received the questionnaire either by mail or via an online link. The same satisfaction questionnaire was used, supplemented with questions about the utilization of recommended interventions and the barriers encountered. For each recommended intervention, patients were asked to indicate the level of implementation (1 = fully to 4 = not (yet)). Perceived barriers were collected using free‐text format.

#### 2.3.2 Data Reported by Clinicians

During the consultation, the following data were recorded: cancer‐related data (cancer diagnosis, disease status, and treatment status) and CRF‐related data (e.g., clinical CRF diagnosis, criteria according to the Fatigue Coalition [24], duration of CRF). Additionally, the documentation form recorded treatment options that were jointly agreed upon by the patient and the clinician.

Based on the patients' responses to the preconsultation questionnaires and their clinical examination findings and medical history, clinicians determined the severity of CRF and classified it as “severe,” “moderate,” “mild,” or “none,” with the latter indicating that the patient did not meet the criteria for a clinician‐based CRF diagnosis. Patients who did not meet criteria for a clinician‐based CRF diagnosis were nevertheless provided with counseling according to their individual needs and, if appropriate, referred to other supportive services available within the counseling centers. As the present analyses focused on CRF‐specific recommendations, their implementation, and satisfaction with the CRF consultation service, only patients with a clinician‐based CRF diagnosis were included in these evaluations.

#### 2.3.3 Data Obtained From Cancer Counseling Centers

Counseling centers collected information on structural and service‐related characteristics of the CRF consultation. These included the number of patients (appointments scheduled and attended), consultation duration, type of consultation setting, and the number of CRF‐specific follow‐up contacts per patient.

### 2.4 Statistical Analyses

Descriptive statistics were reported according to the scale level of the variables: absolute (*n*) and relative (%) frequencies, mean (m) with standard deviation (sd), and median (med) with first and third quartiles (IQR).

A qualitative content analysis [25] was conducted to evaluate the reported barriers to implementing the recommended interventions as well as patients' suggestions for improving the CRF counseling services in the future.

To assess agreement between clinician‐based CRF diagnosis and patient‐reported fatigue as measured by the BFI, as well as between clinician‐based CRF diagnosis and CRF classification according to the Fatigue Coalition criteria, Cohen's kappa statistics were calculated. In addition, one‐way analyses of variance (ANOVAs) were conducted to compare mean scores of fatigue (BFI), depression (PHQ‐9), and anxiety (GAD‐7) across different clinician‐based CRF severity groups.

All statistical tests were conducted using SPSS (Version 29.0.1) at a 5% significance level. Given the exploratory nature of the analyses, no adjustments for multiple testing were applied.

## 3 Results

### 3.1 Utilization of CRF Consultation Service

**FIGURE 1:**
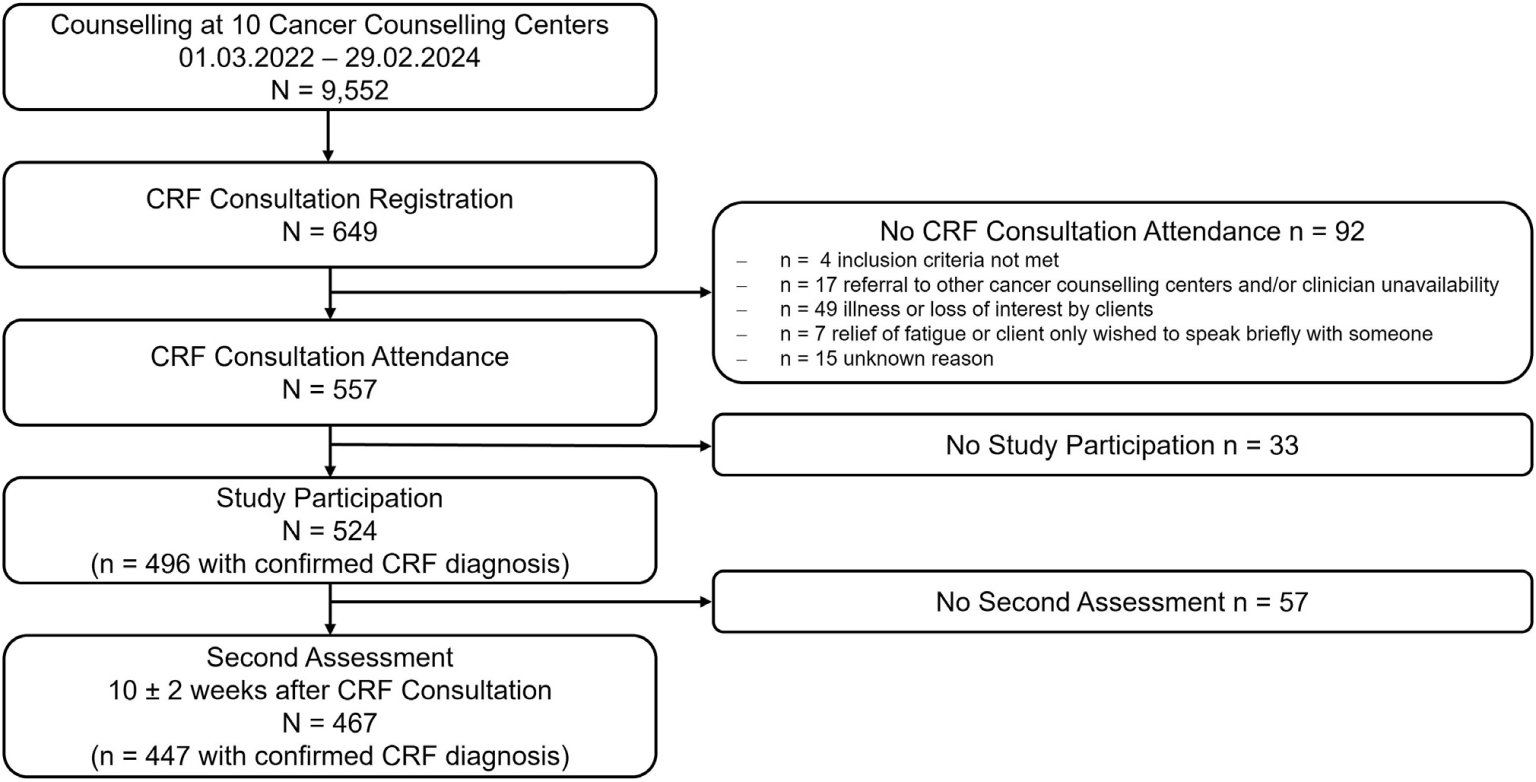
Flow diagram. CRF = Cancer‐related fatigue.

Over a 2‐year period, a total of 9552 patients received counseling at the 10 cancer counseling centers. During this time, 649 patients registered for a CRF consultation, of whom ultimately 557 attended. Of the 557 patients who received CRF consultation, 524 (94%) participated in the study (Figure 1). A total of 467 participants (89%) completed the second survey. The average time between the consultation and the second assessment was 11 ± 2 weeks (range: 5–27 weeks). Table 1 summarizes provision of CRF consultation service.

**TABLE 1:** Provision of cancer‐related fatigue consultation.

*N* = 557 patients attending CRF consultation *N* = 524 patients participating in the study	*n* (%) or mean ± sd (range)
Waiting list	
Yes	132 (23.7%)
No	425 (76.3%)
Time from registration to consultation (days)	54 ± 37 (0–289)
First appointment rescheduled	
Yes	65 (11.7%)
No	492 (88.3%)
CRF‐specific follow‐up contact	
Yes	135 (24.2%)
No	384 (68.9%)
Missing	38 (6.8%)
Number of CRF‐specific follow‐up contacts per client	
0	384 (68.9%)
1	97 (17.4%)
2	16 (2.9%)
3	11 (2.0%)
4	3 (0.5%)
5	1 (0.2%)
6	3 (0.5%)
7	1 (0.2%)
8	1 (0.2%)
9	1 (0.2%)
10	1 (0.2%)
Missing	38 (6.8%)
Total number of CRF‐specific follow‐up contacts	235
Participation in study	
Yes	524 (94.1%)
No	33 (5.9%)
*N* = 524 patients participating in study	
Access pathway	
Cancer counseling center (Bavarian Cancer Society)	251 (47.9%)
Treating clinician (e.g., oncologist)	75 (14.3%)
Psycho‐oncological service/psycho‐oncologist/psychotherapist	37 (7.1%)
Rehabilitation	27 (5.2%)
Patient event/information session	11 (2.1%)
Support group	13 (2.5%)
Flyer/brochure (Bavarian Cancer Society)	77 (14.7%)
Internet (e.g., Bavarian Cancer Society website, online forum)	51 (9.7%)
Media (newspaper/TV)	43 (8.2%)
Social services office	6 (1.1%)
Family/acquaintances	26 (5.0%)
Other	6 (1.1%)
Missing	7 (1.3%)
Consultation setting	
In‐person	487 (92.9%)
Telephone	32 (6.1%)
Online	5 (1.0%)
Consultation duration (minutes)	66 ± 10 (35–105)
Completed second survey	
Yes	467 (89.1%)
No	57 (10.9%)
Time to second survey (weeks)	11 ± 2 (5–27)
Second survey format	
Postal	283 (60.6%)
Online	184 (39.4%)

*Note:* A distinction is made between total patient group attending CRF consultation (*n* = 557) and study cohort (*n* = 524), as some service‐related data were only collected for the latter.

Abbreviation: CRF = cancer‐related fatigue.

Consultations were conducted by 12 clinicians across 10 centers, with one physician per center, except for one center in which the service was provided by three clinicians. During the study period, several centers experienced temporary interruptions in CRF consultations due to clinician unavailability (e.g., illness or vacation). Despite these staffing shortages, close collaboration among the centers enabled client referrals to alternative locations when necessary.

Of the 557 patients attending the CRF consultation, 132 (24%) were initially placed on a waiting list. The average time between registration and consultation was 54 ± 37 days (range = 0–289 days). Sixty‐five clients (12%) missed their first appointment and were offered a rescheduled appointment.

One‐quarter of the patients (24%, *n* = 135) received at least one CRF‐specific follow‐up contact (range = 1 to 10 follow‐up contacts), resulting in a total of 235 CRF‐specific follow‐up contacts. These included inquiries regarding recommended interventions, provision of short written medical statements (“Stellungnahme”), and second consultations.

The following information refers to study participants (*n* = 524). Most patients became aware of the CRF consultation through the cancer counseling centers themselves (48%), followed by a flyer (15%), and their treating clinician (14%). The majority of consultations were conducted in person (93%, *n* = 487). The average consultation duration was 66 ± 10 min (range: 35–105 min); one consultation was conducted over two visits due to further diagnostic assessment.

### 3.2 Characteristics of Study Participants

The mean age of participants was 55 ± 11 years (range = 19 to 89 years), and most were women (77%, *n* = 402). Breast cancer was the most common diagnosis (57%, *n* = 299). At study enrolment, 218 (42%) participants were undergoing ongoing cancer treatment. Participant characteristics are summarized in Table 2.

**TABLE 2:** Participant sociodemographic and clinical characteristics.

*N* = 524 study participants	*n* (%) or mean ± sd (range)
Sex	
Male	116 (22.1%)
Female	402 (76.7%)
Missing	6 (1.1)
Age (years, *n* = 518)	55 ± 11 (19–89)
Educational level^a^	
Low	172 (32.8%)
Medium	148 (28.2%)
High	194 (37.0%)
Missing	10 (1.9%)
Employment status	
Full‐time	83 (18.5%)
Part‐time	188 (35.9)
Due to cancer‐related fatigue	121
Unemployed	244 (46.6)
Due to cancer‐related fatigue	132
Missing	9 (1.7%)
Number of cancer diagnosis	
1	460 (87.8%)
2	53 (10.1%)
3	11 (2.1%)
Cancer diagnosis (multiple answers possible)	
Breast	299 (57.1%)
Lymphatic, hematopoietic, and related tissues	78 (14.9%)
Genital organs	62 (11.8%)
Digestive organs	60 (11.5%)
Respiratory and intrathoracic organs	23 (84.4%)
Thyroid and other endocrine glands	18 (3.4%)
Eye, brain, and parts of the central nervous system	17 (3.2%)
Urinary system	16 (3.1%)
Lip, oral cavity, and pharynx	13 (2.5%)
Other	13 (2.5%)
Ongoing cancer treatment	
Yes	218 (41.6%)
Treatment type (multiple answers possible)	
Antihormonal therapy	147
Chemo−/immuno‐/targeted/antibody therapy	72
Radiotherapy	10
Other	9
Missing	4
No	304 (58.0%)
Missing	2 (0.4%)

^a^ The educational level was classified according to the International Standard Classification of Education 2011.

### 3.3 Diagnosis of CRF

Of the 524 participants, 496 (95%) were diagnosed with CRF by the consulting clinician. In the 5% of cases without a clinical diagnosis of CRF (*n* = 28), differential diagnostics suggested alternative causes for exhaustion such as depressive disorders or immune‐related side effects.

Most participants (88%, *n* = 460) met the diagnostic criteria for CRF as proposed by the Fatigue Coalition. In contrast, 37 (7%) individuals did not meet these criteria, and in 26 (5%) cases, the diagnosis remained uncertain.

The mean BFI score was 6.5 ± 1.5 (range = 1.1 to 9.8), indicating severe fatigue. The mean PHQ‐9 score was 14 ± 5 (range = 1 to 27), indicating a moderate level of depressive symptoms. Mean GAD‐7 was 10 ± 5 (range = 0 to 21), indicating a moderate level of anxiety symptoms.

The cancer‐related fatigue and psychosocial characteristics of the study participants are summarized in Table 3.

**TABLE 3:** Participant cancer‐related fatigue and psychosocial characteristics.

*N* = 524 study participants	Total	Clinician‐based CRF diagnosis^a^			
		None (*n* = 28, 5.3%)	Mild (*n* = 51, 9.7%)	Moderate (*n* = 290, 55.3%)	Severe (*n* = 155, 29.6%)
Fatigue (BFI) (m = 6.5, sd = 1.5, range = 1.1–9.8)		5.5 (95% CI = 4.7–6.3)	5.0 (95% CI = 4.5–5.5)	6.3 (95% CI = 6.1–6.4)	7.7 (95% CI = 7.5–7.9)
Mild	24 (4.6%)	5 (17.9%)	11 (21.6%)	8 (2.8%)	0 (0%)
Moderate	221 (42.2%)	14 (50.0%)	27 (52.9%)	162 (55.9%)	18 (11.6%)
Severe	276 (52.7%)	8 (28.6%)	12 (23.5%)	120 (41.4%)	136 (87.7%)
Missing	3 (0.6%)	1 (3.6%)	1 (2.0%)	0 (0%)	1 (0.6%)
Depression (PHQ‐9) (m = 14, sd = 5, range = 1–27)		11.3 (95% CI = 9.2–13.4)	10.2 (95% CI = 9.1–11.4)	13.4 (95% CI = 12.9–13.9)	16.7 (95% CI = 15.9–17.4)
Minimal	9 (1.7%)	3 (10.7%)	3 (5.9%)	3 (1.0%)	0 (0%)
Mild	88 (16.8%)	8 (28.6%)	19 (37.7%)	51 (17.6%)	10 (6.5%)
Moderate	189 (36.1%)	9 (32.1%)	18 (3.3%)	118 (40.7%)	44 (28.4%)
Moderately severe	157 (30.0%)	5 (17.9%)	9 (17.6%)	91 (31.4%)	52 (33.5%)
Severe	75 (14.3%)	2 (7.1%)	1 (2.0%)	23 (7.9%)	49 (31.6%)
Missing	6 (1.1%)	1 (3.6%)	1 (2.0%)	4 (1.4%)	0 (0%)
Anxiety (GAD‐7) (m = 10, sd = 5, range = 0–21)		8.6 (95% CI = 6.4–10.9)	7.2 (95% CI = 5.8–8.6)	9.5 (95% CI = 9.0–10.0)	12.0 (95% CI = 11.2–12.8)
Minimal	84 (16.0%)	9 (32.1%)	18 (35.3%)	48 (16.6%)	9 (5.8%)
Mild	164 (31.3%)	7 (25.0%)	17 (33.3%)	98 (33.8%)	42 (27.1%)
Moderate	162 (30.9%)	5 (17.9%)	12 (23.5%)	94 (32.4%)	51 (32.9%)
Severe	110 (21.0%)	6 (21.4%)	4 (7.8%)	49 (16.9%)	5 (32.9%)
Missing	4 (0.8%)	1 (3.6%)	0 (0%)	1 (0.3%)	2 (1.3%)
Diagnostic criteria for CRF defined by the fatigue coalition					
Met	460 (87.8%)	6 (21.4%)	37 (72.5%)	265 (91.4%)	152 (98.1%)
Not met	37 (7.1%)	17 (60.7%)	8 (15.7%)	11 (3.8%)	1 (0.6%)
Unclear	26 (5.0%)	5 (17.9%)	6 (11.8%)	13 (4.5%)	2 (1.3%)
Missing	1 (0.2%)	0 (0%)	0 (0%)	1 (0.3%)	0 (0%)

Abbreviations: BFI = Brief Fatigue Inventory, CRF = cancer‐related fatigue, GAD‐7 = Generalized Anxiety Disorder Scale‐7, PHQ‐9 = Patient Health Questionnaire‐9.

^a^ Clinician‐based CRF diagnosis was based on a comprehensive anamnesis that considered various aspects, such as duration, severity, and frequency of CRF, laboratory diagnostics/medical history, and differential diagnostic screenings for anxiety and depression.

Screening scores (BFI, PHQ‐9, GAD‐7) differed significantly across clinician‐based CRF diagnoses (*p*‐values < 0.001). Severity of fatigue symptoms, depression, and anxiety increased with higher levels of clinically diagnosed CRF (*p*‐values ≤ 0.002). No significant differences in fatigue, depression, or anxiety scores were found between participants with no CRF and those with mild CRF (*p*‐values > 0.050).

Agreement between clinician‐based CRF diagnosis (none, mild, moderate, severe) and fatigue severity as measured by the BFI (none, mild, moderate, severe) was 59% (309/521), with a quadratic weighted kappa of 0.31 (95% CI: 0.26 to 0.36; *p* < 0.001). When the CRF was dichotomized (yes/no), agreement between clinician‐based diagnosis and BFI screening increased markedly to 95% (494/521). Agreement between clinician‐based CRF diagnosis (yes/no) and CRF classification based on the proposed diagnostic criteria of the Fatigue Coalition (met, not met, unclear) was high (91%, 471/523), with a kappa of 0.39 (95% CI: 0.28 to 0.51; *p* < 0.001).

### 3.4 Recommendation and Implementation of Interventions

For the patients diagnosed with CRF (*n* = 496), the recommendations and implementation of interventions are summarized in Table 4.

**TABLE 4:** Recommendation and implementation of interventions.

*N* = 496 study participants with clinician‐based CRF diagnosis, t1	*n* (%) or mean ± sd (range)
Number of recommended interventions (total number *n* = 1371)	
1	18 (3.6%)
2	81 (16.3%)
3	397 (80.0%)
Recommended interventions (multiple answers possible)	
Physical activity	310 (62.5%)
Energy conservation and activity management	224 (45.2%)
Mind–body interventions	195 (39.3%)
Psycho‐oncological and psychosocial support	119 (24.0%)
Sleep management	104 (21.0%)
Rehabilitation	80 (16.1%)
Light therapy	69 (13.9%)
Cognitive training	65 (13.1%)
Fatigue reduction diet	62 (12.5%)
Acupuncture/acupressure	55 (11.1%)
Self‐management program “Ways Out of Fatigue”	48 (9.7%)
Cognitive behavioral therapy	24 (4.8%)
*P. ginseng*	12 (2.4%)
Self‐management app “Untire”^a^	4 (0.8%)
Other pharmacological interventions (methylphenidate, bupropion, dexamethasone)	0 (0%)
*N* = 447 study participants with clinician‐based CRF diagnosis, t2	
Implementation of recommended interventions	
At least one^b^	427 (95.5%)
None^c^	20 (4.5%)
Implementation level of recommended interventions (*n* = 1236)	
Fully	87 (7.0%)
Largely	451 (36.5%)
Rather less	394 (31.9%)
Not (yet)	262 (21.2%)
Missing	42 (3.4%)
Barriers implementing the recommended intervention	
Yes	391 (87.5%)
Type of barriers (multiple answers possible)	
Disease‐related	288
Personal and occupational	136
Service‐related	122
Other	129
No barriers reported	56 (12.5%)
Implementation of not recommended interventions	
Yes	172 (38.5%)
Type of not recommended intervention (multiple answers possible)	
Physical activity	49
Clinical treatments/medication adjustments/diagnostic evaluations/alternative therapies and medications	37
Energy conservation and activity management	31
Mind–body interventions	28
Diet	12
Psycho‐oncological and psychosocial support	12
Acupuncture/acupressure	7
Cognitive training	6
Rehabilitation	6
Sleep management	5
Application (e.g., disability pension, rehabilitation)/registration (e.g., patient events, rehabilitation exercise programs)	5
Missing	3
No	266 (59.5%)
Missing	9 (2.0%)

^a^ The app “Untire” was removed from the list shortly after the start of the study, as it became accessible only via a paid access code.

^b^ The level of active involvement in recommended intervention ranged from “fully,” “largely,” to “rather less.”

^c^ Twenty patients implemented none of the recommended intervention. However, 7 of the 20 patients pursued alternative interventions.

In most cases, the maximum of three interventions was recommended (80%, *n* = 397). In summary, 1371 interventions were selected in the context of shared decision‐making with the patients. The interventions recommended most often were physical activity (63%, *n* = 310), energy conservation and activity management (45%, *n* = 224), and mind–body approaches (39%, *n* = 195).

A total of 447 (90%) out of 496 participants completed the follow‐up survey; 20 (4%) out of these 447 patients indicated that they did not initiate any of the recommended interventions (“not [yet]”). The remaining patients (96%) reported that they had started with at least one recommendation. However, the level of active involvement was variable and could be rated as either “fully,” “largely,” or “rather less.” A closer inspection of Table 5 reveals that 44% of the total 1236 recommendations were implemented either “fully” or “largely.”

**TABLE 5:** Implementation level of recommended interventions.

			Implementation					Total
			Fully	Largely	Rather less	Not (yet)	Missing	
Recommended intervention	Physical activity	*n*	17	120	110	24	4	275
		%	6.2%	43.6%	40.0%	8.7%	1.5%	100%
	Energy conservation and activity management	*n*	7	101	74	4	8	194
		%	3.6%	52.1%	38.1%	2.1%	4.1%	100%
	Mind–body interventions	*n*	6	53	73	44	3	179
		%	3.4%	29.6%	40.8%	24.6%	1.7%	100%
	Psycho‐oncological and psychosocial support	*n*	25	34	17	28	4	108
		%	23.1%	31.5%	15.7%	25.9%	3.7%	100%
	Sleep management	*n*	8	42	37	9	4	100
		%	8.0%	42.0%	37.0%	9.0%	4.0%	100%
	Rehabilitation	*n*	5	8	7	48	4	72
		%	6.9%	11.1%	9.7%	66.7%	5.6%	100%
	Light therapy	*n*	4	16	9	33	1	63
		%	6.3%	25.4%	14.3%	52.4%	1.6%	100%
	Cognitive training	*n*	4	18	21	14	4	61
		%	6.6%	29.5%	34.4%	23.0%	6.6%	100%
	Fatigue reduction diet	*n*	2	26	15	10	3	56
		%	3.6%	46.4%	26.8%	17.9%	5.4%	100%
	Acupuncture/acupressure	*n*	4	9	4	32	1	50
		%	8.0%	18.0%	8.0%	64.0%	2.0%	100%
	Self‐management program “Ways Out of Fatigue”	*n*	0	15	19	5	1	40
		%	0.0%	37.5%	47.5%	12.5%	2.5%	100%
	Cognitive behavioral therapy	*n*	3	7	5	6	2	23
		%	13.0%	30.4%	21.7%	26.1%	8.7%	100%
	*P. ginseng*	*n*	2	2	1	5	1	11
		%	18.2%	18.2%	9.1%	45.5%	9.1%	100%
	Self‐management app “Untire”	*n*	0	0	2	0	2	4
		%	0.0%	0.0%	50.0%	0.0%	50.0%	100%
Total		*n*	87	451	394	262	42	1236
		%	7.0%	36.5%	31.9%	21.2%	3.4%	100%

*Note:* For the analysis, only study participants who were diagnosed with cancer‐related fatigue by a clinician and who completed a second assessment were included (*n* = 447). Implementation levels of 1236 recommended interventions are presented.

Even interventions that appear highly relevant to fatigue management and were recommended to over 100 patients were rarely fully or largely followed by more than half of them: physical activity (50%), energy conservation and activity management (56%), mind–body interventions (33%), psycho‐oncological and psychosocial support (55%), and sleep management (50%) (Table 5).

Therefore, not surprisingly, a total of 391 patients (87%) reported barriers to implementing the recommended interventions. Reported reasons were classified as disease‐related (e.g., somatic symptoms, 64%, *n* = 288), personal and occupational (e.g., caregiving responsibilities, 30%, *n* = 136), or service‐related (e.g., lack of service availability, 27%, *n* = 122).

### 3.5 Satisfaction With CRF Consultation

After the consultation, most patients rated the CRF consultation positively. On a rating scale ranging from 1 (very good) to 6 (very poor), the mean rating was 1.2 (range = 1 to 3) immediately after the CRF consultation, and 1.4 (range = 1 to 5) 10 ± 2 weeks after the consultation. A detailed evaluation of the CRF consultation, including 10 single satisfaction aspects, is presented in Table 6. Almost all patients expressed the wish that the CRF consultation will remain available in the future.

**TABLE 6:** Satisfaction with cancer‐related fatigue consultation.

	T1 (*n* = 496)	T2 (*n* = 447)
	*n* (%) or mean ± sd (range)	*n* (%) or mean ± sd (range)
Overall satisfaction rating	1.2 ± 0.4 (1–3)	1.4 ± 0.7 (1–5)
1 (very good)	385 (77.6%)	279 (62.4%)
2 (good)	94 (19.0%)	134 (30.0%)
3 (satisfactory)	3 (0.6%)	21 (4.7%)
4 (fair)	0 (0%)	2 (0.4%)
5 (poor)	0 (0%)	2 (0.4%)
6 (very poor)	0 (0%)	0 (0%)
Missing	14 (2.8%)	9 (2.0%)
Ten consultation‐specific satisfaction aspects^a^	Full/mostly agree	
The consultation should be offered in the future as well	491 (99.0%)	441 (98.7%)
I believe that the consultation is a good resource	491 (99.0%)	438 (98.0%)
The conversation was conducted with empathy and understanding	491 (99.0%)	443 (99.1%)
The explanations were clear and understandable	490 (98.8%)	437 (97.8%)
The consultation was competent and knowledgeable	489 (98.6%)	438 (98.0%)
The consultation was helpful/useful for me	488 (98.4%)	420 (94.0%)
I had enough time available during the consultation	485 (97.8%)	428 (95.7%)
All the important topics were discussed	481 (97.0%)	421 (94.2%)
The waiting time for the consultation was acceptable	465 (93.8%)	417 (93.3%)
I now have a better understanding of fatigue than before	461 (92.9%)	407 (91.1%)
Suggestions for improvements at t1 and t2 (*n* = 271, multiple answers possible)^b^		
Framework conditions of the consultation		157 (57.9%)
Routine follow‐up appointments		65
Consultation duration too short		32
Reduction of waiting times		29
Increase public awareness to make the consultation more widely known		16
Offer more locations		7
More consultation hours with greater time flexibility		3
Other (e.g., parking spaces, air conditioning, quiet rooms)		35
Need for additional support		119 (43.9%)
More information (guidelines, support in implementing the recommendations)		46
Support with clinician/therapist search and administrative matters (e.g., scheduling appointments for patients, assistance with rehabilitation applications)		31
Reducing consultation load (e.g., minimizing the complexity of questionnaires and information)		22
More individualized approach to the patient's life situation		12
Other		43

^a^ For 10 single consultation‐specific satisfaction aspects, “full” and “mostly” agreements were combined and are presented; categories for “somewhat” or “strongly” disagreement as well as missing responses are not presented.

^b^ Patients might have named the same suggestions for improvement both immediately after the consultation and again at follow‐up.

Open‐ended questions to suggest improvements were provided at t1 and t2. Taking both assessments together, a total of 271 suggestions for improvement were provided (Table 6). These suggestions primarily focused on improving the framework conditions (58%, *n* = 157), such as offering follow‐up appointments, reducing waiting times, or extending the duration of individual consultations. Additionally, there was a strong indication that more support was needed (44%, *n* = 119), such as assistance in implementing the recommended interventions, referral to therapists, or help with navigating administrative processes.

## 4 Discussion

The present study yielded four key findings: (1) a higher‐than‐expected demand for consultation service, (2) a high number of confirmed CRF diagnoses, (3) the overwhelmingly positive evaluation of the counseling service, and (4) the varying degrees to which patients follow CRF management recommendations. We discuss these findings in the following sections.

While fatigue management approaches have been described in various health care settings [1, 11], there is currently very limited evidence regarding the evaluation of outpatient CRF consultation services. In particular, data on service utilization, implementation of recommendations, and patient satisfaction are largely lacking [4, 7, 9, 16]. Consequently, direct comparisons of the present findings with existing literature remain limited.

The demand for CRF consultations considerably exceeded the initial expectation of 400 consultations over a 2‐year period. During this project, 557 CRF‐related consultations were conducted, highlighting the urgent need for this kind of service in the German healthcare system.

Diagnostic procedures within the cancer counseling centers followed a multimodal approach that included patient‐reported screening tools, CRF diagnostics, and clinical judgment. In 95% of the patients, the CRF diagnosis could be confirmed, indicating that the screening criteria used to direct patients to CRF consultations were applied effectively. Clinician‐based CRF diagnoses were consistent with patient‐reported BFI and the Fatigue Coalition criteria. Given the absence of a single diagnostic gold standard for CRF, this finding supports the multimodal diagnostic approach used within the consultation service.

A central element of the CRF counseling service was a shared decision‐making process in which patients and clinicians jointly selected up to three evidence‐based interventions to alleviate fatigue. At follow‐up, patients reported on the extent to which they had implemented these recommendations. Only a small proportion of patients (4%) did not initiate any interventions, and 96% initiated at least one. However, the level of active involvement varied between “fully,” “largely,” and “rather less.” Overall, 44% of the recommended interventions were implemented either “fully” or “largely.” Notably, even interventions that appeared highly relevant in the context of fatigue management, such as physical activity or energy conservation and activity management, were not implemented by the majority of patients. Barriers to implementation—which were categorized as disease‐related, personal or occupational, or service‐related—were reported by 87% of the patients. These findings underline that shared decision‐making, while important for patient‐centered care, is not sufficient on its own. Practical and structural support is necessary to translate selected interventions into real‐world behavior.

Despite these challenges, patient satisfaction with the service was exceptionally high, reflected by mean ratings of 1.2 and 1.4, respectively, on a six‐point scale (1 = very good; 6 = very poor). The vast majority of respondents advocated for the continuation of the consultation service, underscoring its perceived value and relevance within supportive oncologic care. Suggestions for improvement primarily focused on structural aspects of the service and the desire for more comprehensive support.

The patient‐reported suggestions for improvement and identified implementation barriers need to be considered within the broader context of current structural limitations in fatigue care. Several of these requests, such as regularly scheduled follow‐up appointments or structured implementation support, extend beyond the scope of a consultation‐based service model. This reflects the well‐documented gap between evidence‐based recommendations and their implementation in routine oncology care. Although CRF is a frequent and clinically significant problem, it remains insufficiently addressed in oncology care and healthcare systems, despite clear guideline recommendations [4]. International and national guidelines recommend routine screening, patient education, and multimodal, evidence‐based treatment [1, 13, 14]. However, their translation into clinical practice and institutional services remains limited [4, 26, 27].

A recent international review confirms low adherence to CRF guidelines among healthcare professionals and a lack of structured integration into care pathways [27]. In Germany, this low level of adherence is echoed by findings from the LIFT study. While verbal information on CRF is commonly provided in oncology institutions, the study revealed that only about one‐third use standardized screening tools, and just one in five offer structured support services [4].

According to existing literature, Certified Comprehensive Cancer Centers (CCCs), in which psycho‐oncological support is mandatory under the German Cancer Society's certification criteria, generally perform better in fatigue care [28]. However, significant disparities persist between institutions and regions. Smaller hospitals, outpatient oncology practices, and rural areas frequently lack the resources or infrastructure to provide systematic CRF management [4].

In line with structural disparities between institutions, literature also identifies a lack of knowledge and training among healthcare professionals regarding CRF treatment options and referral structures as a major barrier to guideline implementation [26, 28]. Outpatient cancer counseling centers offer low‐threshold support, especially during the transition from acute care to long‐term survivorship. The structured decentralized Bavaria‐wide CRF consultation service developed by the Bavarian Cancer Society thus represents a rare example of a specialized, regionally accessible outpatient service that could serve as a best practice model for other regions.

A key structural limitation remains the absence of a dedicated ICD (International Classification of Diseases) code for CRF [29]. Without such classification, CRF‐related services are not eligible for reimbursement by statutory health insurers in Germany. To secure long‐term access to care, policy changes are needed to integrate CRF as a formally recognized condition within healthcare systems. Future expansion of the service will require sustainable funding and long‐term strategic planning.

Several limitations of the present study should be considered. Most participants were women diagnosed with breast cancer. While this group may be particularly receptive to counseling services, it also highlights the need to ensure that other patient groups are equally aware of and able to access these services.

Another limitation of the study is that the data on active involvement in implementing recommendations were based on self‐reports. Our results show that, on average, self‐reported actual engagement is moderate. This moderate engagement may be due to certain structural shortcomings within the current care system, motivational barriers on the part of the patients, or inaccuracies in reporting. These insights are relevant for both improving services and designing future randomized trials: the successful implementation of interventions requires active support and close monitoring to ensure adherence and effectiveness.

## 5 Conclusion

Cancer‐related fatigue (CRF) is a highly prevalent condition that significantly impairs quality of life and remains a major unmet need in cancer care. To address this issue, a structured, evidence‐based CRF consultation service across 10 sites in Bavaria was implemented, with overwhelmingly positive feedback from patients. We believe that this best practice model may serve as a blueprint for nationwide and international implementation.

## Author Contributions

**K. Müller:** conceptualization, investigation, writing – original draft, methodology, validation, visualization, writing – review and editing, formal analysis, project administration, data curation. **M. Besseler:** funding acquisition, conceptualization, writing – review and editing. **T. Pukrop:** writing – review and editing. **J. U. Rüffer:** methodology, writing – review and editing. **J. Weis:** methodology, writing – review and editing. **M. E. Heim:** writing – review and editing, methodology. **I. Fischer:** conceptualization, writing – review and editing, formal analysis, data curation, methodology. **M. Koller:** supervision, data curation, methodology, conceptualization, writing – review and editing, writing – original draft.

## Funding

This study is financially supported by the Bavarian Ministry of Family, Labor and Social Affairs (VI6/33476/45/20).

## Ethics Statement

The study received ethical approval from the Ethics Committee of the University of Regensburg (No. 22‐2796‐101).

## Consent

The study was conducted in accordance with ethical standards. All participants provided written informed consent prior to inclusion in the study. Participation was entirely voluntary, and patients who declined participation were still able to attend the consultation without any disadvantages in terms of care or counseling.

## Conflicts of Interest

The authors declare no conflicts of interest.

## Supporting information


**File S1:** cnr270638‐sup‐0001‐Supplement File S1.docx.
**Figure S1:** Cancer‐related fatigue consultation—standard procedure at the Bavarian Cancer Society (based on Fischer et al. 2016).

## Data Availability

The data that support the findings of this study are available from the corresponding author upon reasonable request.
