# Supplementary material for: Evaluation of an Outpatient Consultation Model for Cancer‐Related Fatigue by the Bavarian Cancer Society: Service Utilization and Patient Satisfaction
Source: Cancer Rep (Hoboken). 2026 Aug 23;9(8):e70638. doi: 10.1002/cnr2.70638 (PMC13500802; doi:10.1002/cnr2.70638)
Supplement: Supplementary file 1 [file CNR2-9-e70638-s001.docx]

**Supplementary File S1 Cancer-Related Fatigue Consultation – Standard Procedure at the Bavarian Cancer Society**

The Bavarian Cancer Society is a non-profit organization that operates 13 cancer counselling centers and 26 outreach locations across the Federal State of Bavaria. The centers provide approximately 30,000 counselling sessions annually. The Cancer-Related Fatigue (CRF) consultation service was introduced in 2013 at a single site, subsequently expanded, and has been available at ten sites since 2021. From the outset, this outpatient consultation service has adhered to the most recent versions of relevant CRF guidelines (e.g., NCCN, ESMO, CAPO), is grounded in evidence-based practice, and is regularly updated to incorporate the latest scientific evidence.

Initial contact is established via the regional cancer counselling centers. Trained psycho-oncologists clarify basic eligibility criteria, such as place of residence (Bavaria), cancer diagnosis, and the presence of exhaustion or fatigue symptoms, and arrange appointments.

The consultation itself is free of charge and conducted by clinicians specially trained in CRF. It typically lasts ~60 minutes and includes standardized diagnostics using a structured anamnesis guideline from the German Fatigue Society and Screening tools such as the Brief Fatigue Inventory. The structured anamnesis covers CRF symptoms and intensity, onset and course, comorbidities (e.g., depression), cancer history, medication, and social background.

Based on the findings, patients receive individualized counselling on CRF, potential contributing factors, further diagnostic steps, and evidence-based treatment options. Shared decision-making guides the selection of suitable interventions.

Non-pharmacological treatment options within the Bavarian Cancer Society include e.g. physical activity (e.g., walking), mind-body interventions (e.g., yoga), and psychosocial support. For pharmacological treatments or causal management of comorbidities, patients are referred to their attending clinicians.

Patients and their attending clinicians may contact the cancer counselling centers as needed, for instance, to discuss how to implement the recommended interventions or coordinate follow-up care. Internal guidelines of the cancer counselling centers permit a maximum of five follow-up sessions per patient per half-year.

This consultation model is systematically documented and may serve as a transferable blueprint for supportive CRF care in other regions.

Supplementary Figure S1 summarizes the standard procedure. For detailed information on the CRF consultation service of the Bavarian Cancer Society see the following articles:

Fischer I, Riedner C, Bojko P et al. Consultation Program for Patients with Cancer-Related Fatigue: A Systematic Evaluation of the Experiences of the Bavarian Cancer Society. Oncol Res Treat 2016; 39: 646–651.

Fischer I, Rüffer JU, Besseler M et al. Tumor-Fatigue-Sprechstunde der Bayerischen Krebsgesellschaft e.V.: Was passiert da eigentlich? Teil I: (Differenzial-)Diagnostik. Forum 2017; 32: 64–67.

Fischer I, Rüffer JU, Besseler M et al. Tumor-Fatigue-Sprechstunde der Bayerischen Krebsgesellschaft e.V: Was passiert da eigentlich? Teil II: Beratung. Forum 2017; 32: 160–163.

**Registration and Appointment Allocation**

by a trained psycho-oncologist

for Bavarian residents with a confirmed cancer diagnosis and symptoms of exhaustion/fatigue

**Cancer-Related Fatigue Consultation**

by trained clinicians operating in an interdisciplinary team

at ten sites in Bavaria

**Individualized Information and Counselling about**

**(Differential) Diagnostics**

Structured anamnesis including e.g., medical history, current laboratory data (if available), CRF-related data, symptoms of anxiety and depression

**Cancer-Related Fatigue and possible contributing factors/causes**

**Evidence-based Treatment Options**

**Further Procedures**

**Treatment options within cancer counselling centres**

- Physical activities
- Mind-Body interventions
- Energy conservation and activity management
- Psycho-oncological and psychosocial support

**Treatment options outside cancer counselling centres**

In consultation with attending clinician:

- Treatment of contributing factors
- Phytopharmaca / psychostimulants

**Supplementary Figure S1 Cancer-Related Fatigue Consultation – Standard Procedure at the Bavarian Cancer Society** (based on Fischer et al. 2016)
